# Architecture and Conservation of the Bacterial DNA Replication Machinery, an Underexploited Drug Target

**DOI:** 10.2174/138945012799424598

**Published:** 2012-03

**Authors:** Andrew Robinson, Rebecca J Causer, Nicholas E Dixon

**Affiliations:** School of Chemistry, University of Wollongong, Australia

**Keywords:** DnaB, DnaC, DnaE, DNA polymerase IIIC, DnaG primase, helicase.

## Abstract

New antibiotics with novel modes of action are required to combat the growing threat posed by multi-drug resistant bacteria. Over the last decade, genome sequencing and other high-throughput techniques have provided tremendous insight into the molecular processes underlying cellular functions in a wide range of bacterial species. We can now use these data to assess the degree of conservation of certain aspects of bacterial physiology, to help choose the best cellular targets for development of new broad-spectrum antibacterials.

DNA replication is a conserved and essential process, and the large number of proteins that interact to replicate DNA in bacteria are distinct from those in eukaryotes and archaea; yet none of the antibiotics in current clinical use acts directly on the replication machinery. Bacterial DNA synthesis thus appears to be an underexploited drug target. However, before this system can be targeted for drug design, it is important to understand which parts are conserved and which are not, as this will have implications for the spectrum of activity of any new inhibitors against bacterial species, as well as the potential for development of drug resistance. In this review we assess similarities and differences in replication components and mechanisms across the bacteria, highlight current progress towards the discovery of novel replication inhibitors, and suggest those aspects of the replication machinery that have the greatest potential as drug targets.

## INTRODUCTION

The overuse of antibiotics during the past 60 years has exerted strong selective pressure on pathogenic bacteria, driving many to develop effective mechanisms of drug resistance [1]. Among the most notorious examples are methicillin-resistant strains of *Staphylococcus aureus* (MRSA) and vancomycin-resistant *Enterococcus* spp. (VRE), both Gram-positives. An equal or perhaps greater threat, however, comes from Gram-negative bacteria like *Acinetobacter* and *Pseudomonas *spp., some strains of which are multi- or even pan-drug resistant [2-4]. Resistant bacteria have developed diverse strategies to evade antibiotic therapy and most worryingly, appear to be developing resistance against an ever-widening spectrum of antibiotic compounds [5]. There is thus an urgent need for the development of new antibiotics with entirely new modes of action to treat infections caused by these highly resistant bacteria [1, 2, 6-8]. Unfortunately, the development of novel antimicrobial compounds has all but ceased in recent years, in part because existing antibiotics were so effective prior to the widespread dissemination of drug resistant strains [2, 9]. Efforts to develop entirely novel antibiotics have been hampered by the inherent difficulty of discovering appropriate cellular targets and functional lead compounds. Most antibiotics developed in recent years have been simple modifications of older compounds, aimed primarily at circumventing problems with resistance [10]. 

The past decade has seen an explosion of data that greatly enhance our knowledge of bacterial physiology [11-17]. High-throughput genome sequencing initiatives have generated more than 1000 complete bacterial genomes [18]. Many hundreds more are near completion. High-throughput gene knockout studies have been used to determine the essentiality of each individual gene in 14 different bacterial species [14]. For well-studied model organisms, such as *Escherichia coli*, large-scale attempts are being made to map the entire cellular protein-protein interaction network [19]. Structural genomics initiatives have now determined three-dimensional structures for many hundreds of bacterial proteins [15]. In addition to these high-throughput studies, researchers using more traditional approaches have made many exciting discoveries in recent years. A highlight is the use of fluorescence microscopy to study the actions of individual proteins inside living bacterial cells, which has added clarity to support decades of *in vitro* studies [20]. Crucially, the data derived from genome sequencing and other high-throughput studies now allow us to extrapolate much of the information derived from traditional work with model organisms to other bacteria, including species that act as human pathogens [21]. 

Are there new opportunities for the discovery of novel antibiotic compounds buried within all these new data? Now is an ideal time to collate this information and use it to assess which among cellular processes might serve as useful targets for drug discovery studies. In general, the biological targets of antibiotics are: (i) essential for growth and propagation of bacterial cells, (ii) conserved across a wide range of human pathogens, and (iii) not present, or distinct from corresponding processes, in humans. Promisingly, there remain some cellular systems in bacteria that satisfy these criteria, yet are not the targets of any current antibiotics. These systems might therefore include new targets for the rational design or discovery of novel antibiotic compounds.

The replication of chromosomal DNA is one such process. It is one of the most fundamental processes carried out by bacteria, yet currently only one functional class of antibiotics (the DNA gyrase inhibitors) targets DNA replication, and even then the mode of action is indirect [22]. The mechanisms underlying bacterial DNA replication are now well understood, particularly in *E. coli* [23-25]. DNA replication is carried out by a highly dynamic complex called the replisome, comprised of at least 13 different proteins (Table **1**). Complete replisome complexes from *E. coli* and *Bacillus subtilis* have been reconstituted from individually purified components and are fully functional *in vitro* [26, 27]. Minimal replicases have been assembled for other bacteria, namely the Gram-positive pathogens *S. aureus* [28] and *Streptococcus pyogenes* [29], the Gram-negative pathogen *Pseudomonas aeruginosa* [30] and the hyperthermophile *Aquifex aeolicus* [31]. Three-dimensional structures are now available for nearly all of the individual protein modules and even for some of the replisomal sub-complexes. The majority of protein-protein interactions have been mapped and are being studied in increasingly finer detail [32]. With an abundance of genome sequence data available, we can now extrapolate our understanding of *E. coli* DNA replication to other organisms [21]. 

Could new antibiotics be designed that target conserved aspects of the DNA replication machinery? If so, will it be possible to avoid the development of resistance encountered so often in the past? In this review we summarize current understanding of bacterial DNA replication, use genome sequence data to map the conservation of replication components across the bacteria, summarize recent efforts to develop DNA replication inhibitors and identify unexploited components that are most likely to be useful as targets for drug discovery and rational drug design.

## BACTERIAL DNA REPLICATION

**Fig. (1):**
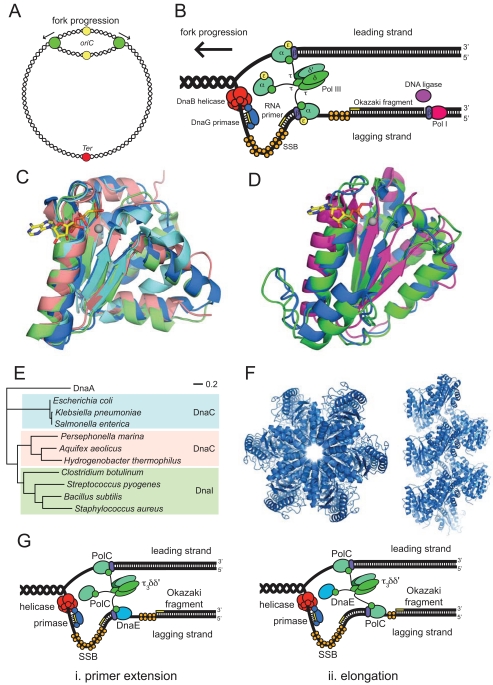
**Architecture and conservation of bacterial replisomes.** (**A**) Bidirectional replication of a circular bacterial chromosome initiates *at oriC* and terminates opposite. Green circles denote replisomes at replication forks. (**B**) Model for leading and lagging strand synthesis at a replication fork in *E. coli.* (**C**) Overlaid ribbon diagrams of the AAA+ domains of DnaA (PDB: 2HCB, blue), DnaC (PDB: 3ECC, green), DnaI (PDB: 2W58, pink) and Hda (PDB: 3BOS, cyan). The position of the ATP analog AMP-PCP (colored by atom type: C, yellow; N, blue; O, red; P, orange) and a Mg^2+^ ion (gray sphere) within the DnaA structure is shown. (**D**) Overlaid ribbon diagrams of the AAA+-like domains of the clamp loader subunits τ (blue), δ (magenta), and δ’ (green). Coordinates were derived from PDB: 3GLI. The positions of ADP (colored by atom type, as above for ATP), the phosphate transition state analog BeF_3_ (Be, magenta; F, cyan) and a Mg^2+^ ion (gray sphere) within the τ subunit are shown. (**E**) Phylogenetic tree based on the sequences of DnaC/DnaI helicase loader proteins. The tree was constructed using the neighborhood-joining tree method in Geneious (Biomatters, Auckland, New Zealand), using the Jukes-Cantor genetic distance model and employing the bootstrap method with 100,000 replicates. The sequence of *E. coli* DnaA was included as an outgroup. Colored boxes indicate helicase loader families (enterobacteria DnaC-type, cyan; Aquificae DnaC-type, pink; firmicute DnaI-type, green). (**F**) Ribbon diagrams showing filaments of *Aquifex aeolicus* DnaC (PDB: 3ECC) formed by P6_1_ crystal packing [49]. (**G**) Model for polymerase handover during lagging strand synthesis in *Bacillus subtilis* [27]. Panels (**C**), (**D**) and (**F**) were created using PyMOL [169].

Although (often) functionally equivalent, the proteins that replicate chromosomal DNA in bacteria (Table **1**) are distinct in sequence and structure from those in eukaryotes and archaea. Despite their enormous genetic diversity, all bacteria appear to share essentially the same mechanisms of chromosomal replication and most of the replication proteins are sufficiently conserved to be readily identified in translated genome sequences. Most bacteria contain a single circular chromosome, within which replication is initiated at a single site, the origin of replication, *oriC* [33]. The two strands of the template DNA are separated at the origin, yielding two fork structures. Replicative DNA polymerases (replicases) and accessory proteins are assembled onto each of these forks, and synthesize new DNA bidirectionally around the circular chromosome (Fig. **1A**) until the two replication forks meet in the terminus region (*Ter*), located approximately opposite the origin. This eventually yields two copies of the bacterial chromosome, each containing one strand from the parental chromosome and one nascent strand.

The best-studied bacterial replication system is that of *E. coli* whose mechanism, for the most part, serves as a model for all bacteria. In *E. coli*, *oriC *is recognized first by the replication initiator protein DnaA, which exists in forms that contain tightly bound ATP or ADP. The origin contains a series of five 9-bp sequence repeats known as DnaA (or R) boxes, to which DnaA-ATP and DnaA-ADP bind, as well as three additional sites (I boxes) that are specific for the ATP-bound form [34, 35]. DnaA appears to remain associated with boxes R1, R2 and R4 for most of the cell cycle. At the onset of a round of DNA replication, binding of ATP-bound DnaA molecules to the remaining sites (R3, I1, I2 and I3) leads to separation of the two template DNA strands at a nearby AT-rich region. Four separate systems regulate this process, ensuring that replication is initiated only once during each cell cycle [34]. Following strand separation, one ring-shaped hexamer of the replicative helicase DnaB (DnaB_6_) is loaded onto each of the DNA strands in the same orientation and each proceeds to unwind the parental DNA duplex, creating replication forks that move away from the origin in opposite directions. The replicase, DNA polymerase III holoenzyme (Pol III HE), associates with the forks and synthesizes both new DNA strands, leaving two completed duplex structures in its wake. Like most DNA polymerases, Pol III cannot begin DNA synthesis on a single-stranded DNA template; it can only extend pre-existing DNA or RNA primers. It is the DnaG primase that first associates with DnaB at the replication fork and constructs short RNA primers, which are then extended by Pol III to build each new DNA strand. 

Following decades of contention, the stoichiometry of individual components within active replisomes has recently been measured in living *E. coli* cells [36]. While DnaG primase was not quantified in this study, existing structural and biochemical evidence indicate that three molecules are likely to be present at each fork [37-39]. Each replisome is comprised of two main sub-complexes: the primosome ([DnaB_6_][DnaG]_3_), and Pol III HE ([αεθ]_3_[τ_3_δδ’(ψχ)][β_2_]_3_)(Fig **1B**). The Pol III HE in turn contains three different subcomplexes: a single clamp loader complex (τ_3_δδ’ψχ) tethers three core polymerases (αεθ), each of which when actively synthesizing DNA is associated with a dimeric β-sliding clamp. Three β_2_ dimers are present at each replication fork [36]; the third could be associated with either the clamp loader or with the third αεθ core. Each Pol III HE thus contains three polymerase cores that are tethered together through the clamp loader complex, and to their DNA substrates by β-sliding clamps. Each polymerase α subunit is accompanied by a proofreading ε subunit, an exonuclease that removes errantly incorporated nucleotides at the 3’ end of the newly synthesized strand. DNA synthesis by Pol III core is carried out only in the 5’→3’ direction; thus synthesis of one nascent strand (the leading strand) is continuous while synthesis of the other strand (the lagging strand) is discontinuous. Synthesis on the lagging strand occurs in four stages. Firstly DnaG primase associates with the DnaB helicase (which translocates on the lagging strand), recognizes a trinucleotide recognition sequence and produces an RNA primer (of up to 14 nt). This happens about once every 1000 nt during lagging strand synthesis. A β-sliding clamp is loaded at the newly primed site by the clamp loader complex, onto which a Pol III core then associates. The Pol III core synthesizes new DNA until it reaches the preceding primer, producing an ~1 kb Okazaki fragment. The Pol III core is then transferred onto a subsequent (upstream) β-loaded primer to begin synthesizing a new fragment. The RNA primers between fragments are removed by ribonuclease HI and/or DNA polymerase I and replaced with DNA by the latter enzyme. Finally the DNA fragments are joined by DNA ligase to create a continuous lagging DNA strand. Throughout DNA synthesis, available template strands are coated by tetramers of the single-stranded DNA-binding protein, SSB, which maintains contact with the replisome through interactions with primase and the χ subunit of the clamp loader. Despite the complexity of this process, DNA synthesis occurs with remarkable fidelity and at an astonishing rate, close to 1000 nt per second [40]. The bidirectionality of chromosome synthesis means that replication of the 4.6 Mb *E. coli* chromosome is completed in about 40 min. Coordinated leading and lagging strand synthesis requires that the replisome be highly dynamic, with components frequently being switched from one part of the template to another. This is made possible through a complex series of protein-protein interactions, some of which are only transient, that coordinate the various enzymatic modules within the replisome assembly.

Three-dimensional structures have now been determined for practically all the functional modules within the bacterial replisome, using proteins from *E. coli* and/or other organisms (Table **1**). The network of protein-protein interactions has been largely determined. Inclusive of initiation complexes, replisome components, SSB, DNA polymerase I and DNA ligase, replication of the bacterial chromosome at the very least requires nine distinct enzymatic activities and ten different protein-protein contacts to be made [32]. These numbers are even greater when essential, but non-universal, components (e.g. helicase loaders) are included. Each of these activities/interactions represents a potential target site for interference by antibiotic compounds. Despite this, very few inhibitors specific for bacterial DNA replication components are available and the replication machinery remains an underexploited target for antibacterial chemotherapy. It is important, however, to consider our knowledge of DNA replication in the context of all bacteria, not just model organisms, if we are to choose DNA replication proteins as targets for drug discovery or rational drug design.

## CONSERVATION OF DNA REPLICATION COMPONENTS IN BACTERIA

With the abundance of bacterial genome sequence data now available, the sequence conservation and phylogenetic distribution of DNA replication proteins is becoming clear (Table **1**). There is a basic replication module that is present in all bacteria and probably arose early in evolutionary history: the DnaA replication initiator, DnaB helicase (called DnaC in firmicutes like *Bacillus *spp.), DnaG primase, Pol III α (plus an additional, related PolC in some organisms), β_2_, ε (part of PolC when PolC is present), τ_3_, δ and δ’, SSB, DNA polymerase I and DNA ligase. This set of proteins constitutes all of the components necessary to carry out and coordinate leading and lagging strand synthesis on a double-stranded DNA template [26, 27]. Other modules are restricted to particular phylogenetic groups and are likely therefore to have evolved more recently, presumably helping to regulate replication events. Some attributes, such as the fusion of ribonuclease HI to the ε subunit of Pol III core [21, 41], have a very restricted distribution (in this case to the family Moraxellacae). Certain other properties, such as the presence of replication termination systems in the Enterobacteriacae (Tus-*Ter* [42, 43]) and firmicutes (RTP-*Ter* [44]), are not essential for cell growth and are therefore of limited interest with respect to drug discovery. Only those components that are essential in those organisms that have them are discussed further. 

In general the sequences of DNA replication proteins are moderately conserved among all bacteria. The genetically distant *E. coli* and *B. subtilis*, for example, share 11–49% sequence identity with respect to the proteins comprising the basic replication module. Overall the sequences of these proteins are less conserved than those of proteins involved in other informational processes such as RNA transcription and protein translation, but more conserved than proteins that function in metabolic pathways and other non-informational processes such as cell division [21]. Within the replication proteins, sequence motifs that encompass enzymatic sites are far more conserved than regions that mediate protein-protein interactions. Similarly, whole proteins that contain enzymatic active sites tend to be more conserved than those that only mediate protein-protein interactions. Notable exceptions to this are the highly conserved protein-protein interaction systems present on the β-sliding clamp and SSB [21, 45, 46]. 

Several DNA replication proteins are paralogs of each other or share structurally related domains. Many belong to the AAA+ family of ATPases, which harness the energy liberated by ATP hydrolysis to perform mechanical functions [47]. Within particular replication sub-complexes the AAA+ proteins are clearly related to each other. The initiator protein DnaA, for example, is closely related to the Hda initiation regulator and (when they are present) the helicase loaders DnaC and DnaI (Fig. **1C**) [48, 49]. Similarly, the τ, δ and δ’ subunits of the clamp loader complex [47], while not all enzymatically active as ATPases, share significant sequence homology and have similar overall structures (Fig. **1D**). These similarities suggest that the members of each sub-complex often share a common ancestor; they presumeably arose through ancient gene duplication events, eventually diverging to take on distinctive roles in the replication process.

Structural homology is also seen among individual domains of some replication proteins. The N-terminal domain of the replicative helicase and the C-terminal domain of DNA primase, which interact to facilitate RNA primer synthesis, share a common fold despite negligible sequence similarity [39, 50, 51]. To date this fold has only been observed in these two proteins. Interestingly, weak sequence homology has recently been revealed for domains of two other replication proteins; the DnaB and DnaD replication initiator accessory proteins found in firmicutes (Table **1**). Using Hidden Markov Model-based homology searches, Marston and colleagues were able to recognise two distinct homologous domains, DDBH1 and DDBH2, within these proteins [52]. DnaD has a DDBH1-DDBH2 domain structure while DnaB includes two DDBH2 domains and is arranged DDBH1-(DDBH2)_2_. Despite their extreme sequence divergence, these domains share common attributes within the two proteins: the DDBH1 domains facilitate subunit tetramerization while the DDBH2 domains bind to DNA (double-stranded DNA for DnaD and the C-terminal domain of DnaB; single-stranded DNA for the first DDBH2 domain of DnaB) and consequently facilitate protein oligomerization.

### Loading of the Replicative Helicase at *oriC*

The process of loading the replicative helicase at the origin has primarily been studied in two model organisms, the Gram-negative enterobacterium* E. coli* and the Gram-positive firmicute *B. subtilis*. In both, the helicase associates with DnaA during loading. In *E. coli*, DnaA-dependent recruitment of DnaB at *oriC* requires the helicase to be in a complex (DnaB_6_[DnaC]_6_) with its loader protein DnaC [53]. DnaC suppresses the ATPase activity of DnaB and remains bound throughout the loading process. Once loaded, primase associates with the helicase, triggering the release of DnaC to enable strand separation by the helicase to commence [54]. Loading of the replicative helicase (called DnaC) in *B. subtilis* follows a similar mechanism except that the helicase loader is called DnaI and the two additional proteins described above, DnaB and DnaD, are also required [55]. Smits and colleagues have recently delineated the order of protein associations at *oriC* that precede helicase loading in this organism [55]. DnaA binds first to *oriC*. The DNA remodeling protein DnaD is then recruited, either through interaction with DnaA or through direct interaction with non-B-form DNA within the DnaA-coated *oriC*. Membrane-associated DnaB molecules are then recruited, probably through direct interaction with DnaD. Finally, two DnaC helicase-DnaI helicase loader complexes associate with the origin, at which point the two helicase hexamers are loaded onto the DNA. The *E. coli* DnaC and *B. subtilis* DnaI loader proteins share significant sequence homology, suggesting that this final step might be somewhat conserved between the two organisms. Interestingly however, relatives of these loader proteins can only be found in a few select groups of organisms outside of the enterobacteria and firmicutes. 

Members of the Aquificae, a small group of hyperthermophilic Gram-negative rods that includes the model organism *Aquifex aeolicus* [56], maintain a helicase loader known as DnaC that is a homolog of *E. coli* DnaC and *B. subtilis* DnaI [49]. As with *E. coli*, no relatives of *B. subtilis* DnaB or DnaD are present in these organisms. For this reason, the Aquificae DnaC proteins have been considered functionally equivalent to *E. coli* DnaC. Phylogenetic sequence analysis demonstrates, however, that Aquificae DnaC proteins are only distantly related to the other DnaC/I helicase loaders (Fig. **1E**). This suggests that Aquificae DnaC proteins represent their own distinct family of helicase loaders and that they might have unique characteristics relative to members of the other two families. 

Collectively, the DnaC/I-type helicase loaders are two-domain proteins, with helicase binding being mediated through the N-terminal domain while ATPase activity and DNA binding occurs within the C-terminal AAA+ domain. Crystal structures of the C-terminal domains of *A. aeolicus* DnaC [49] and *Geobacillus kaustophilus* DnaI [57] reveal very similar overall structures (Fig. **1C**). Sequence homology within this region suggests that the C-terminal domain of *E. coli* DnaC is probably also similar to the equivalent *A. aeolicus *and *G. kaustophilus* domains. The *A. aeolicus *domain was seen to form helical filaments in the crystalline state (Fig. **1F**), reminiscent of filaments previously observed for ATP-bound DnaA from *E. coli* [49]. This helical arrangement is thought to represent a structure important for helicase loading *in vivo* [49]. The fact that the C-terminal domains of helicase loaders are well conserved across the three families suggests that *E. coli* DnaC and *B. subtilis *DnaI probably also adopt helical arrangements during helicase loading. 

The solution structure of the N-terminal domain of *B. subtilis* DnaI determined by NMR, which revealed a novel zinc-binding fold, provides the only structural definition of a helicase loader N-terminal domain [58]. The sequences of the N-terminal domains are conserved within each of the three helicase loader families; however these domains in *E. coli* and *A. aeolicus *DnaCs are clearly unrelated to that of *B. subtilis* DnaI. For both *E. coli* DnaC and *B. subtilis* DnaI, it is this N-terminal domain that facilitates binding to the helicase [53, 59]. Further work is required to determine if the structural basis of the helicase-helicase loader interactions is in any way conserved among the three helicase loader families.

The fact that DnaC/I-type helicase loaders are not conserved among all bacteria implies that organisms outside of the enterobacteria, firmicutes and Aquificae must either not require specialized helicase loader proteins or utilise proteins that are unrelated to DnaC/I for this function [21]. In support of the former scenario, Soni and colleagues have shown that the helicase from *Helicobacter pylori*, an organism that lacks an identifiable helicase loader, can be used to complement a temperature-sensitive *dnaC* mutant of *E. coli* [60]. This implies that the *H. pylori* helicase can be loaded onto the *E. coli* *oriC* using the *E. coli* machinery in the absence of a functional DnaC. One possibility is that DnaA could itself be sufficient for loading of the replicative helicase at *oriC* in organisms that lack helicase loaders. Indeed the helicases from two such organisms, *Pseudomonas* *putida* and *P. aeruginosa* can be loaded onto *oriV* of the RK2 plasmid *in vitro* using only DnaA [61]. In the same system, the *E. coli* helicase required DnaC to be present for loading onto *oriV* [61]*. *Another possibility is that organisms without DnaC/I might utilise replication restart complexes analogous to the PriA/PriB/DnaT system found in *E. coli* [62] to load the helicase at both origin and non-origin sequences. In fact in *B. subtilis*, the DnaB and DnaD proteins are required for loading the helicase both during initiation of DNA replication and for replication restart [63]. Interestingly, homologs of *B. subtilis* DnaB are maintained within members of the tenericutes, which lack homologs of DnaD and DnaI (Table **1**). In this case it is tempting to speculate that DnaB might assist other, as yet unidentified, helicase loaders in loading the helicase at *oriC*.

While helicase loaders are not found in all bacteria, they form an essential part of the DNA replication machinery in those organisms that have them (Table **1**). Importantly, this includes several important human pathogens, such as *E. coli*, *Salmonella typhimurium*,* Bacillus anthracis*, *Listeria monocytogenes*, *Clostridium botulinum*,* Staphylococcus aureus* and *Streptococcus* *pyogenes*. For these organisms, helicase loaders might represent useful antibacterial drug targets. One could imagine, however, that relatively simple modifications to other helicase loading pathways (such as those involved in replication restart) might allow bacteria to survive should their DnaC/DnaI pathways be inhibited. Ultimately this may limit the usefulness of helicase loader inhibitors as antibiotics.

### DnaE1 *versus* PolC + DnaE3 Mechanisms

The majority of bacterial genomes sequenced to date contain a single type of replicative polymerase, the Pol III α subunit, known under the polymerase classification scheme developed by Zhao and colleagues as DnaE1 [64]. It has long been known, however, that the Gram-positive *B. subtilis* uses two related, but distinct, replicase types known as PolC and DnaE3 [27, 64]. DnaE3 is homologous to DnaE1 and has a similar domain organization, while PolC is more distantly related to DnaE1, contains an additional ε-related proofreading domain, and has a dissimilar arrangement of functional domains [64]. Both PolC and DnaE3 are required for DNA replication in *B. subtilis* [65]. Based on the observation that DnaE3 deprivation immediately halts lagging strand synthesis, while allowing leading strand synthesis to continue, it was originally hypothesized that PolC and DnaE3, respectively, constitute separate leading and lagging strand replicases [65], akin to the eukaryotic polymerases Pol ε and Pol δ [66]. Recently however, reconstitution of a functional *B. subtilis* replisome *in vitro* has shown that this is not the case [27].

After identifying 13 proteins likely to comprise the replisome of *B. subtilis* (polymerases PolC and DnaE3, SSB, PriA, DnaG primase, DnaC helicase, DnaB, DnaD, and DnaI helicase loaders, the β clamp, and the clamp loader subunits τ, δ, and δ’), Sanders and colleagues were able to purify each of these components and use them to carry out coordinated leading and lagging strand synthesis on a synthetic minicircle DNA template [27]. All 13 proteins were found to be required for lagging strand synthesis while DnaE3 and primase could be omitted for leading strand synthesis. It was found that both PolC and DnaE3 could extend DNA primers, but only DnaE3 could extend RNA primers like those laid down by primase. Inclusion of both PolC and DnaE3 had a strong synergistic effect on the rate of lagging strand synthesis. These observations are consistent with a model in which DnaE3 does not act as a dedicated lagging-strand polymerase, but rather acts to extend the RNA primers laid down by primase with a short stretch of DNA so that PolC can continue DNA synthesis (Fig **1G**). The role of DnaE3 is thus analogous to that of the eukaryotic Pol α, which extends RNA primers with DNA before handing them over to the lagging strand Pol δ [27]. As PolC/DnaE3 are present in all firmicutes and tenericutes, this model probably applies to all organisms within these groups. 

### Clamp Loader Subunits of DNA Polymerase III

At the heart of the Pol III holoenzyme (minimally [αε]_3_[τ_3_δδ’][β_2_]_2_) is the heteropentameric clamp loader complex (τ_3_δδ’). As its name suggests, this complex functions to load β-sliding clamps onto DNA primer-templates, bestowing leading- and lagging-strand processivity on the replicase [67]. Each of the three τ subunits of the clamp loader also binds to the α subunit of a polymerase core, thus tethering three polymerases together to coordinate synthesis of the two strands [36]. In *E. coli *the *dnaX *gene encoding the τ subunit includes sequence elements that induce a ribosomal frameshift with approximately 50% efficiency [68-72]. This frameshift gives rise to a truncated form of τ, known as γ, comprising the N-terminal three domains out of five in the full-length τ protein; γ can replace τ to form complexes that maintain clamp loading activity, but since it is the C-terminus of τ that contacts α, γcannot multimerize α subunits to form a functional replicase. It has long been thought that the clamp loader complex in *E. coli* contains two τ subunits and one γ, thus yielding a Pol III HE with two polymerase cores ([αεθ]_2_[τ_2_γδδ’][β_2_]_2_). Recently however, the Leake and Sherratt groups have quantified each of the Pol III HE subunits at replication forks in living *E. coli* cells [36], presenting strong evidence that *in vivo*, replicases contain three τ subunits and three αεθ cores. The authors instead propose that clamp loader complexes containing γ subunits could function in other processes that require loading of β-sliding clamps, such as in DNA repair [36]. The programmed ribosomal frameshift encoded within the *E. coli* *dnaX* gene is also conserved in the related organism *Salmonella typhimurium* [73]; in fact similar frameshift sequences are found within *dnaX* genes among all the enterobacteria. The *dnaX* gene of the hyperthermophilic Gram-negative organism *Thermus thermophilus* also produces both τ and γ products [74]. In this case γ is produced by transcriptional slippage rather than a ribosomal frameshift. Most other bacteria, however, do not maintain frameshift signatures within their *dnaX* genes and may not, therefore, produce γ subunits. Indeed the *dnaX* genes from *B. subtilis*, *S. aureus*, *S. pyogenes*, *P. aeruginosa* and *A. aeolicus* produce only full-length τ products when expressed in recombinant *E. coli* systems [27-31, 75]. It appears likely that all bacteria maintain three τ subunits within clamp loader complexes at replication forks and thus utilise trimeric polymerases to replicate their chromosomes.

In addition to τ, δ and δ’ subunits, the clamp loader complexes of γ-proteobacteria include a heterodimeric sub-complex made up of the χ and ψ subunits (Table **1**). This is tethered to the more universal clamp loader assembly (τ_3_δδ’) through an interaction between the N-terminus of ψ and domain III from all three of the τ subunits [76, 77]. The χ subunit interacts with the C-terminus of SSB, providing a mechanism for the handover of primed template DNA from primase to Pol III HE during lagging strand synthesis [78-82]. Recently, the link between SSB and Pol III afforded by the χψ heterodimer has been shown to be crucial for strand displacement activity of Pol III HE [83]. It has also been demonstrated that the interaction of ψ with τ significantly stimulates clamp-loading activity in *E. coli* and *P. aeruginosa* [30, 76, 77, 84]. Interestingly, while only one χψ heterodimer can associate with each clamp loader complex, there are on average four χψ heterodimers at each replication fork in live *E. coli* cells, compared with one τ_3_δδ’ complex [36]. This suggests that additional χψ heterodimers associate with SSB at replication forks, awaiting handover to Pol III HE.

The gene encoding χ, *holC*, is dispensable in most organisms (Table **1**) [14]) and can be disrupted in *E. coli*, although such disruptions show some growth defects [85]. The *holD* gene, which encodes ψ is dispensable in *E. coli* [85] but essential in both *Acinetobacter baylyi* [86] and *P. aeruginosa* [87]. The ψ proteins in these latter organisms are much longer than their *E. coli* counterpart and might therefore carry additional functions that are essential for survival [21, 84]. That the genes encoding χ and/or ψ can be disrupted in *E. coli* and other bacteria suggests that their role in DNA replication is probably regulatory rather than being part of some fundamental mechanism. Interestingly, genes encoding χ appear to be distributed across more of the bacteria (α, β and γ-proteobacteria) than genes encoding ψ (γ-proteobacteria only; see Table **1**). The ψ proteins have highly divergent sequences [21]; thus it is possible that the ψ homologs in β and γ-proteobacteria have simply diverged beyond the detection limit of sequence similarity searches, including sensitive Hidden Markov Model-based methods [88, 89]. If there is a genuine discrepancy in the phylogenetic distribution of χ and ψ subunits, however, this suggests that χ may have evolved to interact directly with τ and/or have extra functions outside of clamp loader complexes.

### Gene Duplications

Genome sequencing has revealed that many bacteria contain genes coding for multiple versions of certain replication proteins. Many of these proteins are potential drug targets and are discussed in detail in the next section. The α subunit of Pol III, which provides the major DNA polymerase activity of the replisome, exists in at least four variants termed DnaE1, DnaE2, DnaE3 and PolC [64]. The first three variants have a similar sequential arrangement of PHP, polymerase and OB-fold domains within their primary sequences, yet can be classified into three different groups using sequence analysis. PolC proteins have regions homologous to the domains found in DnaE-type polymerases, as well as an additional proofreading exonuclease domain. The arrangement of domains is different however, appearing in the primary sequence in the order OB-fold, PHP domain, exonuclease and finally the polymerase domain. As discussed above, all bacteria outside of the firmicutes and tenericutes have DnaE1, while organisms within these groups have both PolC and DnaE3. In contrast, DnaE2 does not conform to phylogenetic boundaries and can co-exist with DnaE1 or PolC and DnaE3. The error-prone DnaE2 variant forms part of a LexA-regulated adaptive mutagenesis cassette, which appears to have originated in the actinobacteria but has since disseminated throughout other bacterial groups through lateral gene transfer [90]. DnaE2 variants are found in the important human pathogens *Mycobacterium tuberculosis* and *P. aeruginosa* and are likely to play a role in the development of antibiotic resistance in these organisms [91, 92].

Other duplications of DNA replication proteins have more mysterious functions. The genomes of many *Bacillus *spp. contain a second copy of the *dnaN* gene, *dnaN-2*, coding for a β-sliding clamp (DnaN-2) that shares approximately 40% sequence homology with its canonical-type β-sliding clamp (DnaN-1) [93]. In *E. coli* the β-clamp is known to bind to the α subunit of Pol III as well as a host of DNA repair enzymes, thus playing a role in both replication and repair [45]. In *B. anthracis*, deletion of *dnaN-2* produces a phenotype indistinguishable from wild-type, whereas deletion of *dnaN-1* results in a mutator phenotype [93]. This, together with the fact that a *dnaN-1*/*dnaN-2* double mutant could not be produced, suggests that DnaN-1 functions in both DNA replication and repair, while DnaN-2 can participate in replication but is deficient in one or more DNA repair functions. 

Duplications of genes encoding the ε subunit of Pol III (*dnaQ*), DNA primase (*dnaG*) and SSB (*ssb*) can also be found within the genomes of a variety of diverse bacteria, not conforming to any obvious phylogenetic boundaries. While some attempts have been made to characterize the products of these duplicated genes, their biological roles remain unclear [94, 95]. Nevertheless, it is important to consider the existence of these gene duplications if the canonical versions of the proteins they encode are to be considered as drug targets, in particular whether any functional redundancy may provide a selective advantage to their hosts when challenged by antibiotics. On the other hand, if two essential proteins with different functions are sufficiently similar to be targeted by the same drug, then this would provide a substantial barrier to selection of resistant mutants in either.

## EXISTING INHIBITORS AND CURRENT SCREENING STRATEGIES

Of all the antibiotics in current clinical use, only two classes inhibit the process of DNA replication. The quinolone and aminocoumarin drugs inhibit the action of DNA gyrase, a type II toposiomerase that introduces negative supercoils in DNA ahead of replication forks to enable continued strand separation by the helicase during DNA replication [96]. Given that the replication machinery includes so many other proteins with functions essential for bacterial viability, it is likely that at least some are useful drug targets. The replisome is very much an under-explored target for drug development, however, and few attempts to discover specific inhibitors have been described. One of the major challenges in discovering DNA replication inhibitors has been the need for biochemical assays that can be used for high-throughput screening. Important advances have been made in this area recently and are likely to yield new inhibitors in the near future. This section describes inhibitors that have been identified so far and the current strategies used for discovering new antagonists of replication activity.

### DNA Gyrase Inhibitors 

Gyrase (a type IIA DNA topoisomerase) plays an essential role in DNA replication, actively underwinding the double-stranded template DNA ahead of the replication fork to obviate effects of positive supercoiling induced by the progressing replisome. It acts by cutting both DNA strands, passing double-stranded DNA through the gap, then religating the original ends [97]. It is a heterotetrameric enzyme comprised of two different subunits: GyrA, which binds DNA and carries out strand cleavage/ligation, and GyrB, which hydrolyses ATP to drive the supercoiling reaction. Gyrase is the cellular target of several different classes of bacteriocidal agents, including the highly-successful fluoroquinolone antibiotics, coumarins and cyclothialidines that are currently undergoing drug development, as well as a range of naturally-occurring bacterial toxins [22, 97]. Much of the current research on gyrase inhibitors focuses on modifying existing scaffolds to address issues with drug resistance, toxic side effects and poor cellular penetration, as well as to broaden the range of bacterial species that compounds are active against [22, 97, 98]. Structure-aided design and virtual screening techniques have proven somewhat successful in delivering new classes of gyrase inhibitors, some of which show broad-spectrum antibacterial activity and reduced rates of resistance relative to exisiting drugs [99, 100]. Most recently, there has been exciting progress with two novel classes of gyrase inhibitors that show great promise for development into new antibiotics in the near future.

**Fig. (2):**
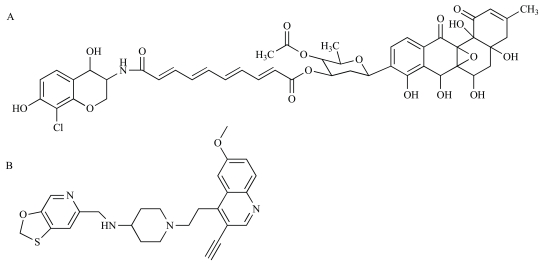
**Chemical structures of new inhibitors of DNA gyrase.** (**A**) Simocyclinone D8 [101-105]. (**B**) GSK299423 [106].

The novel antibiotic simocyclinone D8 was originally identified in extracts from the antibiotic-producing bacterium *Streptomyces* *antibioticus* Tü 6040 [101]. This compound has a chlorinated aminocoumarin group linked to an angucyclic polyketide moiety through a tetraene linker and a D-olivose sugar (Fig. **2A**). The presence of the aminocoumarin group suggested DNA gyrase to be the cellular target and simocyclinone D8 was shown to potently inhibit the *E. coli* enzyme *in vitro* [102]. Unlike the aminocoumarin antibiotics however, simocyclinone D8 was found not to inhibit the ATPase activity of the GyrB subunit, nor did it stimulate the formation of unproductive cleavage complexes within the GyrA subunit as seen with quinolone compounds, suggesting an entirely novel mode of inhibition [102]. Edwards and colleagues have recently determined the crystal structure of simocyclinone D8 in complex with the N-terminal domain of the GyrA subunit, revealing binding pockets for the aminocoumarin and polyketide moieties close to, but distinct from a previously identified quinolone-binding site [103]. This represents a significant step in the development of simocyclinones into clinically useful antibiotics, as structure-based activity relationships can now be explored. In addition to this binding site within the GyrA subunit, biochemical studies have suggested the existence of a second low-affinity binding site within GyrB [104]. Further work is now required to explore the possibility of cooperative binding at the GyrA and GyrB sites. Interestingly, while simocyclinone D8 shows poor activity against Gram-negative laboratory bacteria and is thus being viewed as a potential treatment only for infections caused by Gram-positives, it has recently been found to have potent activity against a number of Gram-negatives isolated from clinical samples [105]. This observation is likely to assist in identifying parameters that can be optimized to broaden the spectrum of activity of simocyclinones, and adds to the promise of these compounds becoming clinically useful antibiotics.

A very promising new antibiotic that acts on gyrase has been recently described by GlaxoSmithKline researchers [106]. GSK299423 (Fig. **2B**) is a member of the novel bacterial topoisomerase inhibitors (NBTI) family of compounds, which has also been explored by Novoxel [107] and Johnson & Johnson [108, 109]. GSK299423 strongly inhibits the activity of *S. aureus *and *E. coli* gyrase *in vitro* (IC_50_ = 14–100 nM), making it more than 2000 times more potent than the widely used drug ciprofloxacin (a fluoroquinolone) [106]. GSK299423 shows potent antibacterial activity against a range of Gram-positive and Gram-negative pathogens (MIC = 0.016–8 µg/mL), including strains that are resistant to fluoroquinolones. The crystal structure of the gyrase-DNA-GSK299423 complex has been determined using the* S. aureus* enzyme, revealing that the compound binds between the active sites of the two GyrA subunits, away from known binding sites for quinolone-type inhibitors [106]. GSK299423 appears to inhibit the catalytic cycle of gyrase by stabilizing a pre-cleavage enzyme-DNA complex, an entirely novel mode of gyrase inhibition. Taken together, the potency of GSK299423, its broad-spectrum activity and its lack of cross-resistance with existing fluoroquinolones suggest the NBTIs represent exciting candidates for development into a much-needed new class of antibiotics with a novel mode of action.

### Inhibitors of DNA Polymerase Activity

DNA polymerases are the targets of several important anti-viral and anti-cancer drugs, yet few inhibitors exist for the equivalent bacterial enzymes [110]. To date, there are no known inhibitors that are specific for DnaE1-type polymerases (e.g. the α subunit of *E. coli* Pol III). Two classes of compounds are known to effectively inhibit the PolC-type polymerases found in the firmicutes and tenericutes: 6-anilinouracils (6-AUs) and quinazolin-2-ylamino-quinazolin-4-ols (BisQuinols). Members of a third class of compounds, the dichlorobenzylguanines, have been shown to inhibit the activities of DnaE1-, DnaE3- and PolC-type polymerases, although no analysis of their antimicrobial properties has been published since their syntheses were described six years ago [111]. 

**Fig. (3):**
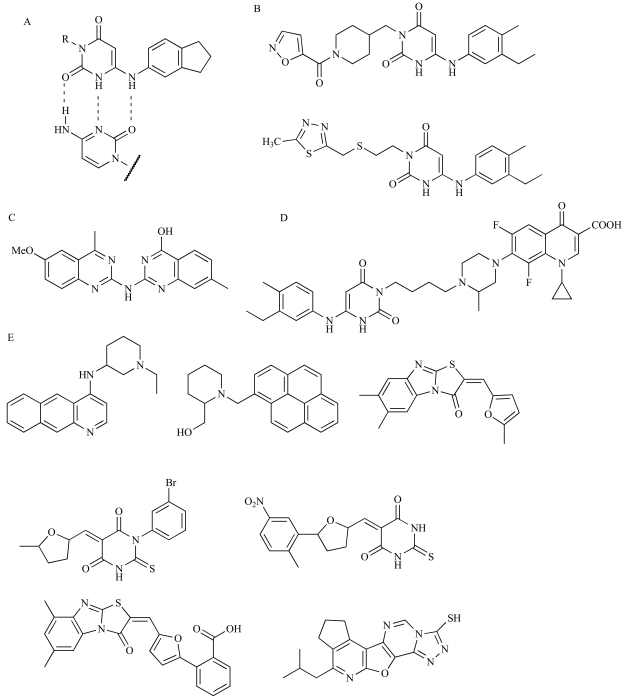
**DNA polymerase inhibitors.** (**A**) Three-hydrogen-bond interaction between 6-anilinouracil compounds and a cytosine residue within DNA [115]. Chemical structures of (**B**) new 6-anilinouracil derivatives with improved aqueous solubility [118], (**C**) a BisQuinol inhibitor of PolC [121], (**D**) a hybrid PolC/DNA gyrase inhibitor [116], and (**E**) novel replisome inhibitors identified by high-throughput screening [127].

The 6-anilinouracils are the oldest and most developed class of DNA polymerase inhibitors [112-114]. These compounds are competitive inhibitors of dGTP binding to PolC [115], forming a three-hydrogen-bond base pair with cytosine residues in the template DNA (Fig. **3A**). Many variations have been made on the original 6-AU scaffold, producing several compounds that potently inhibit PolC activity and bacterial growth *in vitro *[115-119]. Historically however, 6-AUs have proven modest inhibitors of bacterial growth in animal infection models due to poor solubility and bioavailability [120]. Most compounds that have demonstrated activity *in vivo* have been tested using intraperitoneal dosing, which largely circumvents the problems associated with poor sample solubility. Recently, Svenstrup and colleagues addressed this issue by varying 3-substituents on the 6-AU scaffold with a view to improving aqueous solubility, and in doing so have produced two compounds (Fig. **3B**) that show strong activity against *S. aureus *and *Enterococcus faecalis* using intravenous dosing in a mouse infection model [118]. It is hoped that this advance in understanding of the structure-activity relationships of 6-AUs will facilitate the development of clinically useful compounds. 

Members of the second class of PolC inhibitors, the BisQuinols, were discovered recently using high-throughput fluorescence-based inhibition assays [121]. The methodology underlying these assays is described later in this section. It was predicted that BisQuinols might base pair with cytosine residues in DNA templates and thus be competitive with dGTP substrates, as observed for the 6-AUs. Biochemical analysis revealed, however, that BisQuinols were uncompetitive with respect to nucleotide substrates, instead showing competitive behaviour against template DNA. The mechanism of PolC inhibition by BisQuinols must therefore be different to that of 6-AU compounds and appears to be more analogous to that of non-nucleoside reverse transcripttase inhibitors used to treat HIV infections [121, 122]. Importantly, the initial series of BisQuinol compounds also showed significant inhibition of the eukaryotic Pol δ and thus appears to show poor selectivity for bacterial DNA polymerases [121]. Nevertheless, one representative compound (Fig. **3C**) was found to be somewhat effective in rescuing mice from a lethal intraperitoneal *S. aureus* infection, thus demonstrating a degree of *in vivo* efficacy. A better understanding of the structure-activity relationships for this novel class of PolC inhibitors is now required to assess their potential for development into functional antibiotics.

A promising new strategy for the development of PolC inhibitors is to covalently link 6-anilinouracil derivatives to fluoroquinolones to create hybrid PolC-DNA gyrase inhibitors. These hybrid compounds maintain dual anti-PolC and anti-DNA gyrase activity *in vivo* and have greater or equal potency against whole bacterial cells than their isolated 6-AU or fluoroquinolone parent compounds [116, 123, 124]. In fact when testing one representative hybrid (Fig. **3D**), Butler and colleagues observed that the fusion to fluroquinolone actually increased the anti-PolC activity of the 6-AU moiety [116]. Even more promisingly, in cases where each of the parent compounds was only weakly active in isolation, i.e. for drug-resistant organisms, the hybrid compound was found to offer greater potency than an equimolar mixture of its parents. The hybrid compound was thus effective against a broader range of bacterial strains than either parent, an attribute also observed for oxazolidinone-quinolone hybrid protein synthesis/DNA gyrase inhibitors [125, 126]. The use of hybrid inhibitors intuitively should provide greater protection against the development of drug resistance by target mutagenesis than traditional single-target inhibitors – this would require co-mutation of two loci in the bacterial population. This indeed appears to be the case for Butler’s hybrid compound, for which resistance in *S. aureus* was seen to develop far more slowly than resistance to either parent compound in a simple pure culture system [116]. 

Several bacterial replisomes have now been reconstituted and are functional* in vitro* [26-28, 30, 127]. For *E. coli *and *B. subtilis*, this includes all the machinery required for both leading and lagging strand synthesis [26, 27]. For other species, more minimal replisomes capable only of leading strand synthesis have been assembled [28, 30]. Recently, Dallmann and colleagues described a high-throughput assay to screen for inhibitors of reconstituted replicase activity, making use of an intercalating fluorescent dye to monitor conversion of single-stranded DNA substrates into a double-stranded product [127]. This approach has a significant advantage over assays that focus on isolated replication components, in that inhibitors of any part of the replication machinery can be identified through a common endpoint. These workers also included specificity assays to test if compounds bind to DNA, or inhibit viral and eukaryotic DNA polymerases, RNA polymerase, an unrelated eukaryotic ATPase or an unrelated control enzyme, β-galactosidase. Using these assays, they screened a small library of 2000 compounds and identified seven (Fig. **3E**) that act as specific inhibitors of bacterial replisomes and kill bacterial cells by inhibiting DNA replication. This high-throughput assay approach thus provides an efficient means to generate lead compounds for further drug development, and could be applied equally well for screening natural product and commercially available libraries of drug-like compounds. 

### Inhibitors of Primosome Functions

**Fig. (4):**
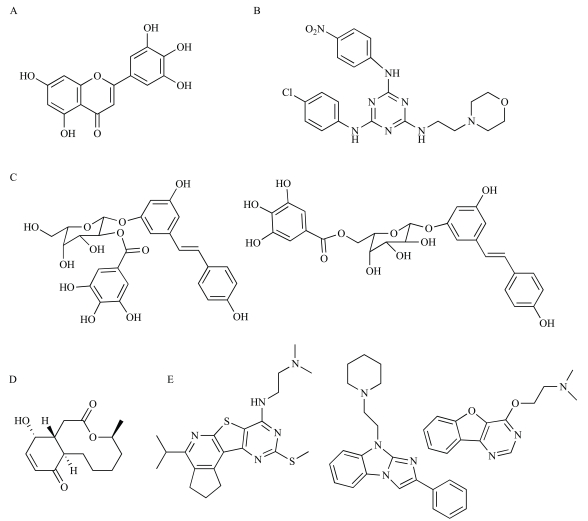
**Chemical structures of inhibitors of primosome functions.** (**A**) The DnaB helicase inhibitor, myricetin [129]. (**B**) A triaminotriazine inhibitor of DnaB helicase [147]. (**C**) Phenolic saccharide inhibitors of DnaG primase [144]. (**D**) Sch 642305, an inhibitor of DnaG primase [146]. (**E**) Inhibitors of DnaG primase produced using structure-aided design [145].

Outside of DNA gyrase and the replicative polymerases, another replication subcomplex to be targeted for drug discovery has been the primosome, comprised of the DnaB/C helicase and DnaG primase (see Table **1**). These proteins play critical roles in lagging strand synthesis and are essential for bacterial growth [40, 85, 128]. These proteins are targets for some naturally occurring DNA replication inhibitors. The dietary flavonoid myricetin (Fig. **4A**), for instance, has antimicrobial activity and has been shown to potently inhibit the replicative helicase, but not primase [129]. The bacterial alarmone compound (p)ppGpp on the other hand, is now known to inhibit the activity of primase, thereby halting DNA replication as part of the stringent response [130, 131]. The existence of these natural, low molecular weight inhibitors lends hope that the primosome subcomplex might be a viable target for drug discovery.

The replicative helicase has two functional domains. The N-terminal domain recruits primase to the replication fork, while helicase activity stems from the C-terminal domain. The helicase domain belongs to the RecA-type family of ATPases [132], which includes the bacterial replication/ recombination/repair proteins RepA [133] and RecA [134], the transcription termination factor Rho [135] as well as the eukaryotic DNA repair proteins Rad51 and DMC1 [136]. On the other hand, primase has a central RNA polymerase domain that is not related to other RNA polymerases and has a uniquely-shaped active site [137]. This domain is flanked by an N-terminal zinc-binding domain, which binds to specific trinucleotide initiation sequences on the template DNA, and a C-terminal domain that binds to the helicase [138]. Primase interacts with the helicase to enable primer synthesis on the lagging strand *in vivo* [32]. This interaction stimulates the activity of both proteins *in vitro* [139] and varies in strength, ranging from a weak and transient complex as observed for the *E. coli* proteins [140] to a highly stable complex as for the *G. stearothermophilus* proteins [37].

In recent years high-throughput biochemical assays have been developed to measure the activities of helicase and primase, as well as the mutually stimulatory effects of their interaction [141-143]. Application of these assays in drug discovery has been limited, however, and has produced only a few inhibitors [144-146]. The Förster resonance energy transfer (FRET) assay for DnaB helicase activity developed by Zhang and colleagues [141], for instance, has been used by researchers at Targanta Therapeutics to screen a library of 230,000 commercially available compounds for inhibitors of* P. aeruginosa* DnaB [147]. The *Pseudomonas* DnaB helicase was used as it can be efficiently loaded onto substrate DNA molecules in the absence of a helicase loader protein, in contrast to the equivalent helicases from *E. coli* and *B. subtilis* [61, 148]. Their screen revealed a triaminotriazine compound that inhibited the helicase with IC_50_ = 5 µM (Fig. **4B**). Despite showing potent inhibition of the *P. aeruginosa* DnaB *in vitro*, neither the newly identified compound nor analogs prepared to explore effects on cytotoxicity were found to inhibit the growth of *P. aeruginosa* cells, despite the fact that a permeability barrier-deficient strain was used [147]. Some compounds were, however, moderately active in inhibiting the growth of *S. aureus*, *S. pneumoniae* and a hyper-susceptible strain of* E. coli*. Unfortunately, this series of compounds exhibited a range of undesirable properties, including cytotoxicity towards HeLa cells and loss of antimicrobial activity in the presence of serum, that ultimately deem them unsuitable as lead compounds for antibiotic development. To date this represents the only reported systematic attempt to identify specific inhibitors of replicative helicase function.

DNA primase has attracted slightly more attention as a drug target, although success has again been limited. To date all searches for inhibitors have utilized the scintillation proximity assay for primase activity developed by Zhang and colleagues [141]. The assay is based on the ability of primase to incorporate radioactive ^3^H-labeled NTP substrates into an RNA product, which is then captured using a biotin tag on the template DNA strand to which the RNA product remains hybridized. Researchers at Schering Plough used this assay to screen extracts of various plants for inhibitors of *E. coli* primase [144], unearthing two novel phenolic saccharides from *Polygonum cusoidatum* that produced IC_50_ values < 5 µM (Fig. **4C**). Unfortunately, these compounds appear to act by binding to the template DNA rather than to primase itself and are therefore not appropriate lead compounds [129, 144]. In another similar study conducted by Schering Plough researchers, a novel bicyclic macrolide compound, Sch 642305 (Fig. **4D**), was isolated from the fungus *Penicillium verrucosum* and found to inhibit *E. coli* primase with an IC_50_ of 70 µM [146]. Promisingly, Sch 642305 also inhibited the growth of the *E. coli* strain HS294 (defective lipopolysaccharide layer and disrupted *acrAB* efflux pump) with an MIC of 40 µg/mL. As yet no attempts have been reported to establish the mode of Sch 642305 inhibition. Presumably this is due to difficulty in obtaining sufficient material for biochemical analysis, although several synthetic approaches for production of Sch 642305 have now been described [149-151]. Before Sch 642305 can be considered as a lead compound, further work is required to determine its mode of action, as well as its efficacy against other bacterial strains in both *in vitro* cell growth and *in vivo* animal infection models.

Making use of the high-resolution crystal structure of the RNA polymerase domain of *E. coli* primase [137], Agarwal and colleagues at Achillion Pharmaceuticals used virtual screening to identify three potentially ‘druggable’ sites within primase [145]. They selected 79 compounds predicted to bind at these sites, and using the scintillation proximity assay [152] identified four inhibitors with IC_50_ values of 7–50 µM [145]. None of these compounds was active in inhibiting the growth of *E. coli* cells, however. The initial four compounds were used to deduce a three-dimensional pharmacophore, which was then used to search a database of commercially available compounds for other potential inhibitors. From this search, a further 34 chemically diverse compounds were selected for enzyme inhibition assays, revealing eight additional compounds that inhibited primase with IC_50_ values < 100 µM. Three of these also inhibited the growth of *E. coli*, with MIC values of 4–64 µg/mL (Fig. **4E**). Analogs of these compounds were then studied with these assays, allowing structure-activity relationships to be deduced for each chemical series [145]. Further work is now required to assess the efficacy of these compounds against other bacterial strains for antibiotic development.

### Inhibitors of the Initiation of DNA Replication

A relatively new approach towards the discovery of DNA replication inhibitors is to target the initiation stage. In (almost) all bacteria, the first step in replicating the chromosome is the binding of multiple DnaA molecules at the origin, which ultimately leads to separation of the two template strands [153]. Since this step signifies commitment to a complete round of replication, the amount and activity of DnaA is tightly regulated within the cell. Insufficient DnaA leads to under-initiation, while excess leads to over-initiation. Both disrupt cell growth. Currently, no antibacterial agents target the initiation process. 

Fossum and colleagues have recently developed a robust cell-based assay to screen for inhibitors of DnaA activity [154]. The assay utilises an *E. coli *strain containing a novel *dnaA* allele that produces an over-active DnaA variant and confers a cold-sensitive phenotype. This strain grows well at the permissive temperature (42˚C), but grows poorly at 30˚C due to excessive initiation of DNA replication. Compounds that inhibit DnaA activity reduce the initiation rate and thus restore cell growth at 30˚C. This was demonstrated by expressing moderate levels of the DnaA N-terminal domain, a competitive inhibitor of full-length DnaA, within the cells. While a small-scale screen of microbial extracts failed to identify any inhibitors of DnaA activity, the simplicity of this assay would easily facilitate larger screens and could lead to the discovery of inhibitors in the future.

A second cell-based assay has been developed recently with the aim of discovering inhibitors of replication initiation in *Vibrio* spp. [155]. This group of bacteria includes the causative agent of cholera, common sources of food poisoning and pathogens of economically important marine animals. Unlike most bacteria, *Vibrio* spp. contain two chromosomes, only one of which is replicated in a DnaA-dependent manner. Initiation of replication at the second chromosome relies on the activity of a second protein, RctB, that bears no sequence similarity to DnaA [156]. Yamaichi and colleagues established a cell-based screen for identifying inhibitors of *V. cholerae* RctB [155]. The assay uses an *E. coli* strain that carries an RctB-dependent plasmid expressing both RctB and a kanamycin-resistance marker. If RctB is inhibited by a compound the cells cannot replicate the plasmid and thus do not grow on kanamycin-containing media. Using this strain, the group screened a library of 138,000 small molecules, identifying a potent inhibitor of RctB, vibrepin, that inhibited the growth of all tested *Vibrio* spp. but had no effect on the growth of wild-type *E. coli* cells. If RctB inhibitors such as vibrepin can be developed into functional antibiotics, they should only kill the infectious *Vibrio* spp. whilst not affecting the natural gut flora of a patient, offering a significant advantage over current treatment options.

## POTENTIAL OPPORTUNITIES FOR DRUG DISCOVERY

### Structure-Aided Design

Despite the fact that three-dimensional structures are known for most bacterial replication proteins, few attempts to design novel inhibitors using structure-aided drug design have been described. Structure-aided design involves *in silico* screening of small molecule libraries, seeking to identify compounds that dock into binding sites on the surface of the target protein [157]. Docked ligands are then expanded to maximize contacts with the protein, to optimise the affinity of the small molecule for the binding site. A major challenge is that X-ray crystal structures, the most common source of structural data for proteins, provide only a structural snapshot (i.e. a single conformation) that might not reflect aspects of flexibility important for the *in vivo* activity of the protein. Structure-aided design does have the advantage that structure-activity relationships can often be easily deduced and important parameters can be rationalized on the basis of the geometry of binding pockets. Historically however, compounds designed this way very often have problems with toxic side effects [158]. Nevertheless, a small number of drugs produced using computer-aided design have made it into clinical use [158, 159]. 

The most common design strategy is to target enzyme active sites within the protein structures [157]. The replication machinery includes DNA and RNA polymerase sites as well as several ATPases. Given the ubiquity of ATP transactions in all living cells, these latter enzymes present a significant challenge for rational design of inhibitors with high specificity. Nonetheless, studies using bacteriophage replicases have shown that it is possible to develop inhibitors with specificity towards certain ATPase active sites over others [160]. Given that many of the ATPases in the bacterial replication machinery are related (Fig. **1B-C**), it might be possible to develop compounds that inhibit multiple essential replication components, yet do not act on unrelated ATPases. The mode of ATP binding has been deduced for all of the replisome-associated AAA+ proteins and key residues comprising binding pockets have been identified [49, 57, 76, 161, 162]. Replicative and non-replicative AAA+ proteins could now be compared to assess similarities and differences in binding sites that could be exploited to design compounds that specifically inhibit bacterial DNA replication.

The high-resolution crystal structure of *G. kaustophilus* PolC in complex with template DNA and dGTP substrate has provided the first detailed view of the active site of a bacterial chromosomal replicase [163]. Residues comprising the active site are highly conserved in the PolC subunits of other firmicutes, including those of several pathogens. The crystal structure of PolC will likely form an excellent model for structure-aided drug design, particularly if further structures can be solved for complexes with 6-anilinouracil-type inhibitors. Unfortunately, due to poor sequence conservation between PolC and DnaE1-type polymerases (e.g. the Pol III α subunit) and the low resolution of structural information for the ternary complex between DnaE1 and DNA [164], it is difficult at the moment to model the DnaE1 active site. The specificity for 6-AUs towards PolC over DnaE1-type polymerases suggests that there are significant differences in architecture of active sites between the two types. Higher-resolution data are required for DnaE1-DNA-substrate complexes if this form of polymerase is to be used in structure-aided design.

The replisome is a highly dynamic complex in which many proteins are tethered to each other by way of flexible linkers. Almost all of the extant structural data has involved truncation of proteins to individual domains or removal of flexible portions. Identifying constructs suitable for structure determination has proven one of the key challenges in the study of replication proteins. Now that crystallizable constructs are available, many are prime candidates for structure-based fragment screening approaches [165]. This strategy involves determining many (often several hundred) high-resolution structures of the protein of interest, after exposing crystals of it to mixtures of drug-like fragments. Fragments found to bind at adjacent sites on the protein surface are then linked, producing a tighter-binding compound that acts as a lead for drug development. Fragment-based screening uses smaller compound libraries compared to other high-throughput screening approaches and has greater hit rates against difficult targets [165]. It is anticipated that if applied to replication proteins, fragment screening could yield novel inhibitors of enzymatic activities and protein-protein interactions that are crucial for replication, and thus provide a starting point for the development of novel antibiotics that target the DNA replication machinery. 

### Protein-Protein Interaction Hubs

The portions of replication proteins that form protein-protein contacts are generally poorly conserved among bacterial genera. There are, however, two protein-protein interaction systems in bacterial replication that are extremely well conserved – those based around the β-sliding clamp and SSB [21]. The β-sliding clamp is known to bind (at least) to DNA polymerases I, II, III, IV and V, DNA ligase, Hda, MutS and MutL in *E. coli*, acting to recruit these proteins to their sites of action on double-stranded DNA during replication and repair processes [21, 45]. As far as is known, each of these proteins binds to the same hydrophobic groove on the β-sliding clamp by way of a pentameric or hexameric peptide motif, often located at or close to the N- or C-terminus [45]. The hydrophobic groove on the β-sliding clamp and its binding motifs in other proteins are strongly conserved across bacterial species [21]. An analogous mechanism is also shared by the PCNA sliding clamp of archaea and eukaryotes, although the consensus sequence recognised by PCNA is different from that of the bacterial β-binding motifs. The strong conservation of this protein binding mechanism across bacteria, together with the essentiality of many of the interactions formed at this single site, makes the protein-binding groove of the β-sliding clamp an attractive target for rational antibiotic design. 

**Fig. (5):**
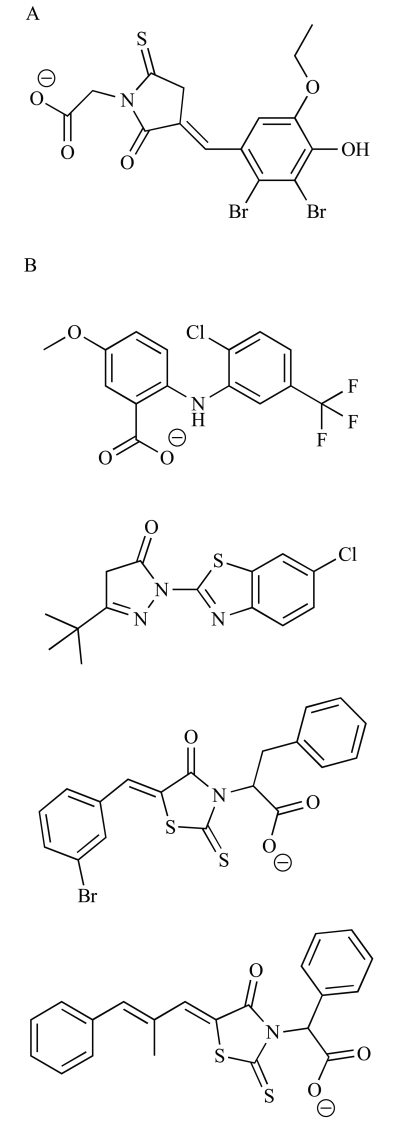
**Chemical structures of protein-protein interaction inhibitors.** (**A**) RU7, an inhibitor of β-sliding clamp interactions [166]. (**B**) Inhibitors of interactions mediated by the conserved Cterminal peptide of SSB [168].

The O’Donnell group has recently developed a high-throughput fluorescence polarization anisotropy assay for identifying inhibitors of β-clamp-DNA polymerase interactions [166]. The assay is based on the displacement of a fluorescent TAMN-labeled peptide, derived from the *E. coli* Pol III α subunit, from its binding site on the β-clamp. From a library of >30,000 small molecules, 91 inhibitors were identified. These were then screened in a DNA replication assay, revealing 19 compounds that specifically inhibited the β-dependent activity of Pol III HE. A subset of these compounds was also active in displacing a PolC-derived peptide from *S*. *pyogenes* β, consistent with the high level of conservation of protein-binding-site residues in β subunits from diverse bacteria [21]. One compound, RU7 (Fig. **5A**), was further capable of differential inhibition of binding of various polymerases to β, inhibiting the β-dependent activities of Pol III and Pol II, but not Pol IV [166]. Importantly, RU7 was found not to inhibit PCNA-dependent DNA synthesis by a model eukaryotic polymerase, yeast Pol δ. The O’Donnell compounds seem to be useful leads that could eventually be developed into a new class of bacterial DNA replication inhibitors.

SSB also interacts with many DNA replication and repair proteins by way of a conserved peptide motif, recruiting them to their sites of action on single-stranded DNA [21, 46]. These interactions are mediated by the final 6–9 residues at the C-terminus of SSB [79], which in *E. coli* has the sequence -MDFDDDIPF. These residues are highly conserved across a wide range of bacterial species, indicating that this system is probably used universally by bacteria. A crystal structure of the terminal SSB peptide in complex with exonuclease I shows the final two residues of SSB bind within a hydrophobic pocket on the exonuclease I surface [167]. It is anticipated that its binding to other proteins occurs in a similar fashion. By employing a fluorescence polarization anisotropy assay similar to that developed for monitoring β-clamp interactions [166], Keck and colleagues screened a library of >50,000 small molecules, identifying four (Fig. **5B**) that disrupt the interaction between the SSB C-terminus and exonuclease I [168]. Of these, two contained groups analogous to the Phe residue at the C-terminus of SSB. Interestingly, these same two compounds inhibited SSB interactions with RecQ and PriA, each structurally unrelated to exonuclease I, demonstrating at least some similarity in the arrangement of SSB binding sites in these proteins. Given that the SSB protein interaction system is conserved in bacteria, but not present in eukaryotes, it may be possible to develop these compounds into selective inhibitors of bacterial DNA replication.

The β-clamp and SSB interaction hubs are promising novel drug targets. If inhibitors of these interactions can be designed, they should be capable of simultaneously disrupting several functions critical to the survival of a bacterial cell. Such inhibitors should also have broad-spectrum activity as these modules are highly conserved. Finally, and perhaps most crucially, the fact that numerous proteins interact at a single site on each of these hubs should help to preclude development of resistance to such inhibitors – development of resistance by target modification would require simultaneous mutation of many different essential sites. The accumulation of point mutations that decrease the affinity of inhibitory compounds for their target is a common route to antibiotic resistance. In the case of protein-protein interaction sites, any mutation occurring on one interacting partner needs compensatory mutation(s) on another to maintain the integrity of the (essential) interaction site. For this to occur in the β-clamp and SSB protein-binding sites, it would necessitate the unlikely acquisition of simultaneous compensatory mutations in the binding sites of many or all of the numerous proteins that bind at these sites. 

## PERSPECTIVES

The lack of antibiotics that target the bacterial DNA replication machinery is perhaps surprising given that it is one of the best characterized and understood of complex biological processes. Unlike many other essential processes, few useful inhibitors of replication proteins have been discovered within natural product extracts. One explanation might be that inhibiting bacterial replisomes is inherently difficult and that even Nature has struggled to develop small molecule inhibitors against them. Until recently, however, suitable assays to test for specific inhibition of the replication machinery have not been widely available, so useful replication inhibitors might simply have gone undiscovered. A series of assays has now been developed that allow for high-throughput screening for inhibitors. Together with an abundance of protein structural data and a solid understanding of the protein-protein interactions present within active replisomes, it is hoped that selective inhibitors can be developed in the near future. We must however, learn from lessons of the past and choose targets that minimize opportunities for the development of drug resistance. One way that this can be achieved is by incorporating genome sequence data into the target selection process, to ensure that only highly conserved components of the replication machinery are marked for drug development. With this in mind, the enzymatic sites found in the replicative DNA polymerases, primase, helicase and various AAA+ proteins, as well as the highly conserved protein interaction hubs formed around the β-sliding clamp and SSB are likely to have the most potential as targets for drug discovery and rational design.

## Figures and Tables

**Table 1.** Bacterial DNA Replication Proteins

Protein	Function	Phylogenetic distribution	Essentiality*	Enzymatic activity†	Interaction partners	Structures‡ (organism)*	References
*Initiation complex*							
DnaA	initiator	all bacteria	Ab, Bs, Ec, Fn, Hi, Mg, Mp, Mt, Pa, Sa	ATPase	DnaB, Hda, DiaA, Dps, HU, DNA	2HCB (Aa), 1L8Q (Aa), 2E0G (Ec), 2Z4R (Tm)	[161, 170-176]
Hda	initiation suppressor	α/β/γ- proteobacteria	Ec, Pa	ATPase	β, DnaA	3BOS (Sh)	[162, 177]
YabA	initiation suppressor	firmicutes	D	–	β, DnaA	–	[178]
DnaB	helicase co- loader	firmicutes, tenericutes	Bs, Sa	–	DnaC (helicase), DnaD, DnaI	–	[55, 179]
DnaC	helicase loader	enterobacteria, Aquificae	Ec, St	ATPase	DnaB (helicase), DNA	3ECC (Aa)	[49, 53, 54]
DnaD	DNA remodelling	firmicutes	Bs, Sa, Sn	–	DnaB, DnaI	2V79 (Bs)	[55, 63, 180]
DnaI	helicase loader	firmicutes	Bs, Sa, Sn	ATPase	DnaC (helicase), DnaB, DnaD, DNA	2K7R (Bs), 2W58 (Gk)	[58, 181]
*Primosome*							
DnaB/DnaC	DNA helicase	all bacteria	Ab, Bs, Ec, Fn, Hi, Mg, Mp, Mt, Pa, Sa, Sn	ATPase	DnaA, DnaC/DnaI, DnaG, Rep, DNA	2Q6T (Ta), 2VYE (Gk), 2R6A (Gs), 1B79 (Ec), 1JWE (Ec), 2R5U (Mt)	[37, 53, 54, 63, 132, 139, 182-186]
DnaG	DNA primase	all bacteria	Ab, Bs, Ec, Fn, Hi, Mg, Mp, Mt, Pa, Sa, Sn	RNA primase	DnaB, SSB, DNA	1D0Q (Gs), 1DDE (Ec), 1EQN (Ec), 3B39 (Ec), 2R6A (Gs), 1Z8S (Gs), 2HAJ (Ec), 1T3W (Ec), 2AU3 (Aa)	[37, 39, 50, 54, 78, 137, 139, 140, 187-189]
*DNA Pol III core*							
DnaE	α subunit, polymerase activity	all bacteria	Ab, Bs, Ec, Fn, Hi, Mg, Mp, Mt, Pa, Sa, Sn	DNA polymerase	ε, β, τ, DNA	2HNH (Ec), 2HPI (Ta), 3E0D (Ta)	[26, 164, 190-194]
DnaQ	ε subunit, proofreading activity	α/β/γ- proteobacteria	Ab, Ec, Fn, Hi, Pa	exonuclease	α, θ, DNA	1J53 (Ec), 2IDO (Ec)	[192, 195-197]
HolE	θ subunit	enterobacteria	D	–	ε	2AXD (Ec), 2AE9 (Ec)	[198-200]
PolC	polymerase activity	firmicutes, tenericutes	Bs, Mg, Mp, Sa	DNA polymerase/ exonuclease	β, τ, DNA	3F2B (Gk), 2P1J (Tm)	[27, 28, 65, 75, 163, 190]
*DNA Pol III clamp loader complex*							
DnaX	τand γ subunits	all bacteria	Ab, Bs, Ec, Fn, Hi, Mg, Mp, Mt, Pa, Sa, Sn	ATPase	α, δ, δ’, γ, ψ, DnaB	2AYA (Ec), 3GLI (Ec), 1NJ5 (Ec), 1XXH (Ec)	[76, 201-204]
HolA	δ subunit	all bacteria	Ab, Bs, Ec, Fn, Hi, Mp, Pa	–	τ, δ’, γ,β	3GLI (Ec), 1XXH (Ec), 1JQL (Ec)	[76, 203, 205]
HolB	δ’ subunit	all bacteria	Ab, Bs, Ec, Fn, Hi, Mg, Mp, Pa, Sa, Sn	ATPase	τ, δ, γ	3GLI (Ec), 1XXH (Ec)	[76, 203]
HolC	χ subunit	α/β/γ- proteobacteria	Hi	–	ψ, SSB	1EM8 (Ec)	[78, 83, 206]
HolD	ψ subunit	-proteobacteria	D	–	χ, τ/ γ	1EM8 (Ec)	[76, 84, 206]
*Other replication proteins*							
DnaN	β sliding clamp	all bacteria	Ab, Bs, Ec, Fn, Hi, Mg, Pa, Sa	–	α, δ, Hda, UmuC, UmuD, DinB1, MutS, MutL, DNA ligase, PolA, PolB, DNA	2POL (Ec), 1JQL (Ec), 2AVT (Sp)	[26, 45, 172, 191, 192, 205, 207-212]
SSB	ssDNA binding	all bacteria	Ab, Bs, Ec, Fn, Hi, Mg, Mp, Pa, Sa, Sn	–	DnaG, χ, RecQ, TopB, UmuC, RecJ, PriA, RecO, exonuclease I, GroEL, DNA	1EQQ (Ec), 1Z9F (Tm), 2VW9 (Hp), 1SE8 (Dr), 1UE1 (Mt), 2FXQ (Ta)	[46, 78, 79, 81-83, 167, 213-225]
PolA	DNA polymerase I	all bacteria	Ab, Bs, Ec, Fn, Hi, Mg, Mp, Mt, Sa	DNA polymerase/ exonuclease	β, DNA	1DPI (Ec), 1KLN (Ec), 1KFS (Ec), 3BDP (Gs), 3KTQ (Ta)	[190, 210, 226-230]
LigA	DNA ligase	all bacteria	Ab, Bs, Ec, Fn, Hi, Mg, Mp, Mt, Pa, Sa, Sn	DNA ligase	β, DNA	2OWO (Ec), 1DGS (Tf), 1B04 (Gs), 1TA8 (Ef), 3JSL (Sa), 1ZAU (Mt)	[210, 231-235]
DNA gyrase	DNA supercoiling	all bacteria	Ab, Bs, Ec, Fn, Hi, Mg, Mp, Mt, Pa, Sa, Sn	topoisomerase ATPase	GyrI, CcdB, DNA	2WL2 (Ec), 2XCQ (Sa), 2XCS (Sa), 2XCT (Sa)	[103, 106]
Tus	terminator	enterobacteria	D	–	DNA	2EWJ (Ec)	[43]
RTP	terminator	some bacillales	D	–	DNA	2EFW (Bs), 1BM9 (Bs), 1F4K (Bs)	[44, 236, 237]

* Organism designations: Aa, Aquifex aeolicus; Ab, Acinetobacter baylyi; Bs, Bacillus subtilis; Dr, Deinococcus radiodurans; Ec, Escherichia coli; Ef, Enterococcus faecalis; Fn, Francisella novicida; Gk, Geobacillus kaustophilus; Gs, Geobacillus stearothermophilus; Hi, Haemophilus influenzae; Hp, Helicobacter pylori; Mt, Mycobacterium tuberculosis; Mg, Mycoplasma genitalium; Mp, Mycoplasma pulmonis; Pa, Pseudomonas aeruginosa; Sa, Staphylococcus aureus; Sm, Shewanella amazonensis; Sn, Streptococcus pneumoniae; Sp, Streptococcus pyogenes; St, Salmonella typhimurium; Ta, Thermus aquaticus; Tf, Thermus filiformis; Tm, Thermatoga maritima; Vc, Vibrio cholerae; D, dispensable; gene not found to be essential in any organism.

†,– no activity or no structure available

‡ Codes shown are Protein Data Bank accession number
